# Dataset on biochemical markers and histological alterations in rat kidney intoxicated with cadmium chloride and treated with antioxidant

**DOI:** 10.1016/j.dib.2022.108394

**Published:** 2022-06-17

**Authors:** Esmaeil Karami, Zahra Goodarzi, Ali Ghanbari, Alireza Dehdashti, Ahmad Reza Bandegi, Sedighe Yosefi

**Affiliations:** a Department of Occupational Health, Engineering, School of Health, Semnan University of Medical Sciences, Semnan, Iran; b Research Center of Physiology, Semnan University of Medical Sciences, Semnan, Iran; c Department of Biochemistry, Faculty of Medicine, Semnan University of Medical Sciences, Semnan, Iran; d Research Center of Health Sciences and Technologies, Semnan University of Medical Sciences, Semnan, Iran

**Keywords:** Atorvastatin, Cadmium chloride, Kidney, Rat model, Biochemistry, Histology, Oxidative stress

## Abstract

This dataset demonstrates the in vivo renal histology and biochemical activity of Atorvastatin (AT) in cadmium-induced nephrotoxic rat model. Fifty-six adult male Wistar rats assigned to eight groups. Rats were treated with physiologic saline at a volume of 4 mg/kg, contained Atorvastatin at a dose of 20 mg/kg body weight for 15 days. The intraperitoneal administration of cadmium chloride at doses of 1, 2, 1 and 3 mg/kg started on day 8. On day 16, samples were collected for biochemical and histological analyses. Data of renal function were estimated in the serum and organ. Cadmium chloride increased malondialdehyde (MDA), blood urea nitrogen (BUN), and creatinine (Cr) serum level and decreased superoxide dismutase (SOD), glutathione peroxidase (GPx) and glutathione (GSH) levels. Administration of Atorvastatin significantly increased lipid peroxidation and renal decreased glutathione and antioxidant enzymes activity and significantly decreased BUN and Creatinine levels. Data were supported by histological examination indicated improved changes and kidney protective potential following cadmium chloride-induced oxidative stress.

## Graphical abstract

**Figure:**
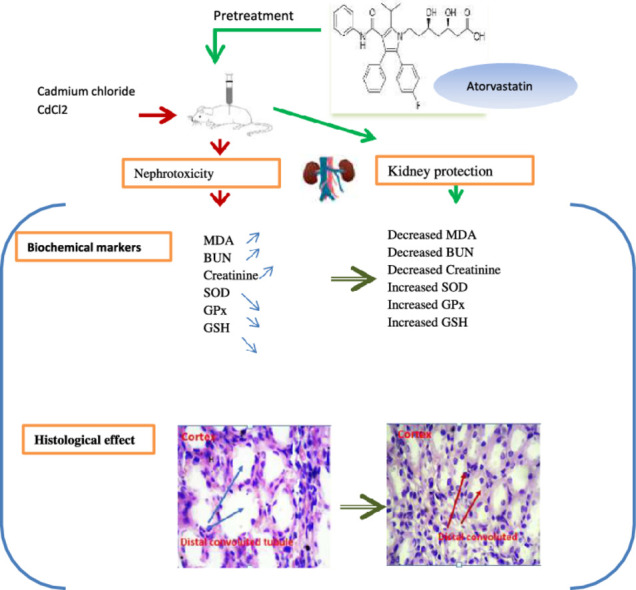

**Specifications Table**

**Table:** 

Subject	Toxicology, Pharmacology
Specific subject area	Environmental and occupational toxicology
Type of data	Text file, Table, image and figures
How data were acquired	Biochemical data were measured using Rat GPX ELISA Kit (Cat No: ZB-GPX-96A), Rat SOD ELISA Kit (Cat No: ZB-SOD-96A), Rat GSH ELISA Kit (Cat No: ZB-GSH-96A), and Rat MDA ELISA Kit (Cat No:ZB-MDA-96A). ELISA kits obtained from Zellbio Germany. BUN and CR in the kidney tissues were measured by Kits obtained from Pars Azmun Pharmaceutical, Tehran, Iran.
Data format	Raw and analysed data
Parameters for data collection	Parameters determined were serum levels of BUN and CR and renal tissues concentrations of MDA, GSA, GPx, and SOD as oxidative stress biomarkers. Histological parameters measured for kidney tissue injury were cell nuclear dilation, loss of staining capacity and cellular swelling.
Description of data collection	Nephrotoxicity was induced in Wistar rat by the administration of cadmium chloride. Atorvastatin was administered for fifteen days. Histology of the kidney tissue and biochemical data were collected to evaluate the effect of Atorvastatin on these parameters in cadmium-induced nephrotoxicity. During each animal examination, the blood was collected from the heart of rats, which was subsequently centrifuged to obtain the serum. Kidneys were dissected, blocked, and for each tissue a 10 μm segment was sectioned and collected. The slides were made and dried and then stained with haematoxylin and eosin (H&E) protocols for histological analysis. Lastly, In the surface of each slide, five fields were randomly selected and evaluated under 400 x magnification by one pathologist and histologist.
Data source location	Semnan University of Medical SciencesSemnanIran
Data accessibility	With the articleRepository name: MendeleyDirect URL to data: https://data.mendeley.com/datasets/fknzy9twbz/3
Related research article	Goodarzi, Z.; Karami, E.; Yousefi, S.; Dehdashti, A.; Bandegi, A.R.; Ghanbari, A. Hepatoprotective effect of atorvastatin on Cadmium chloride induced hepatotoxicity in rats. Life Sci. 2020, 254, 117770, doi:10.1016/j.lfs.2020.117770.

**Value of the Data**

• The dataset presents favourable evidence concerning the effect of Atorvastatin treatment on oxidative stress biomarkers (MDA, SOD, GPx, and GSH) and histological structure data in cadmium chloride-induced nephrotoxicity in Wistar rat model.
• The antioxidant activity data of Atorvastatin at the dose of 20 mg/kg body weight in rats intoxicated with cadmium chloride provides researchers with reference for further experimental studies
• The data supported Atorvastatin protective effect in cadmium-induced kidney intoxication in rat model and therefore, may be used for further experiments in investigating the potential Atorvastatin preventive effect against kidney damage in workers who are occupationally exposed to Cadmium.
• These data indicate Atorvastatin improves lipid peroxidation, BUN, and Creatinine serum levels and reduces adverse histological changes in rat kidney tissues induced by cadmium chloride.
• The insights gained from these data may allow researchers explain the role of Atorvastatin treatment on the cadmium chloride- induced oxidative stress in rat nephrotoxicity.

## 1 Data description

The current data article describes the effect of Atorvastatin treatment on oxidative stress biochemical markers and histological changes in rat nephrotoxicity induced by cadmium chloride.

**Fig. 1:**
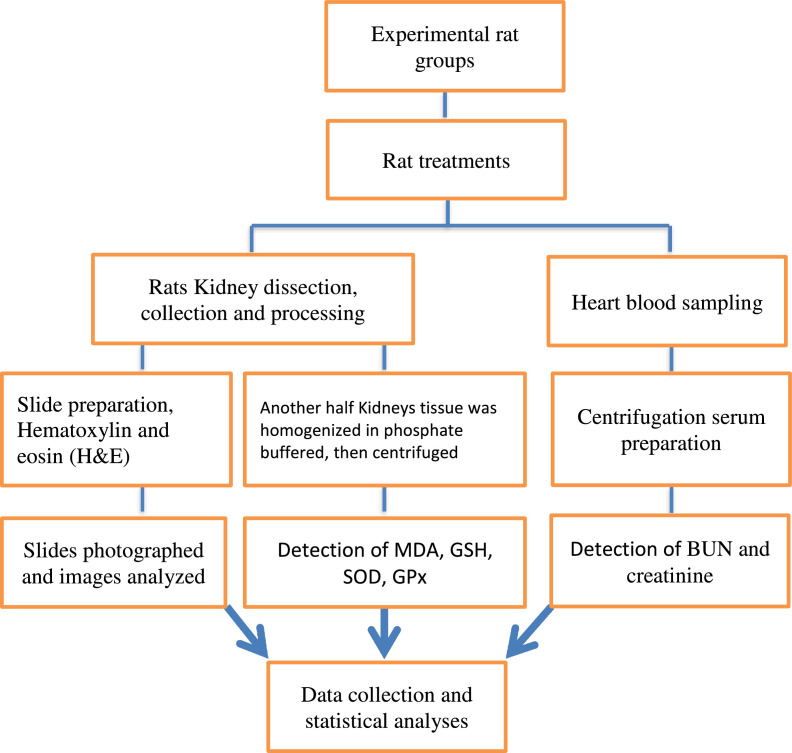
Experimental Steps to obtain biochemical and histological data in rats kidney.

Fig. 1 presents the experimental steps that were conducted to collect data on biochemical and histological parameters.

**Fig. 2:**
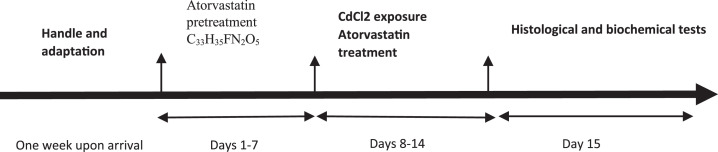
Experimental rat treatments.

**Fig. 3:**
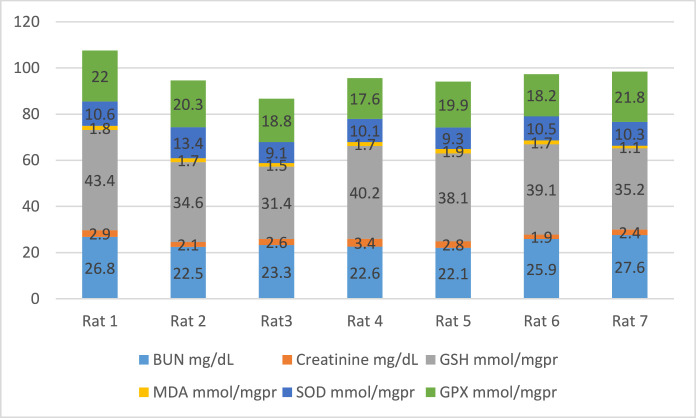
Data on kidney biochemical markers for group 1 treated with physiologic saline.

**Fig. 4:**
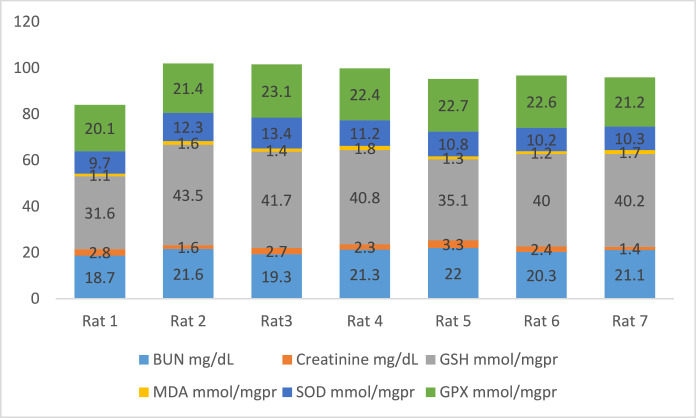
Data on kidney biochemical markers for group 2 treated with Atorvastatin at a dosage of 20 mg/kg/day for 15 days.

**Fig. 5:**
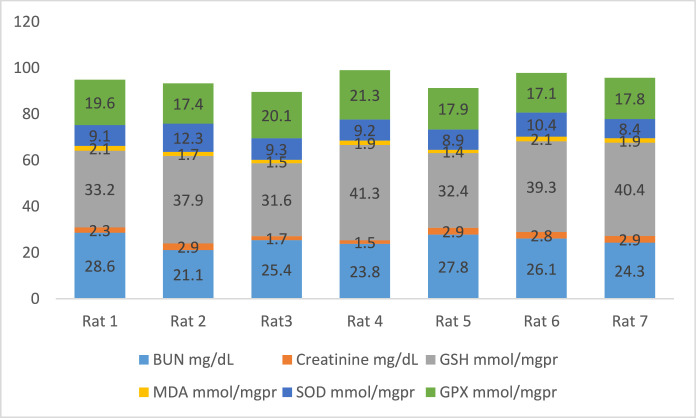
Data on kidney biochemical markers for group 3 treated with CdCl2 at a dosage of 1 mg/kg body Weight.

**Fig. 6:**
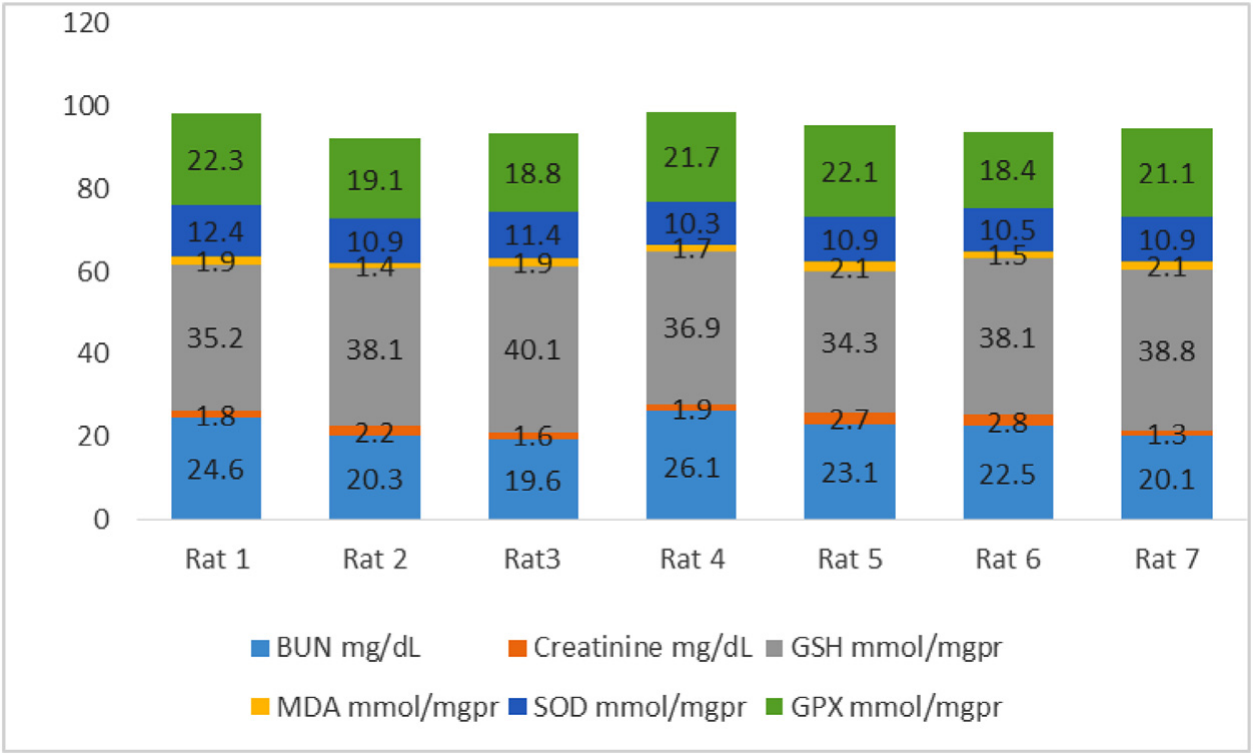
Data on kidney biochemical markers for group 4 treated with Atorvastatin (20 mg/kg body weight) and CdCl2 (1 mg/kg body weight).

**Fig. 7:**
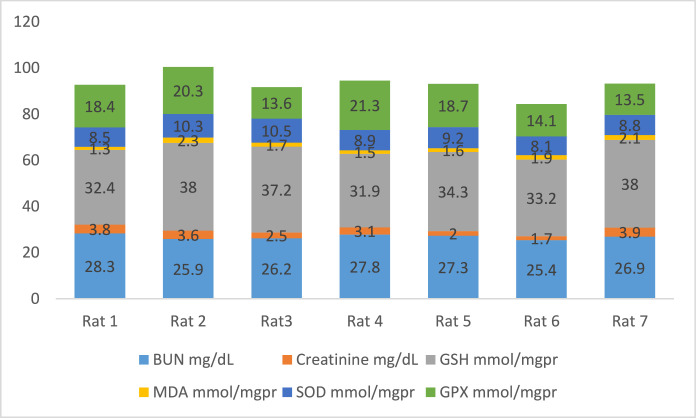
Data on kidney biochemical markers for group 5 treated with CdCl2 (2 mg/kg body weight).

**Fig. 8:**
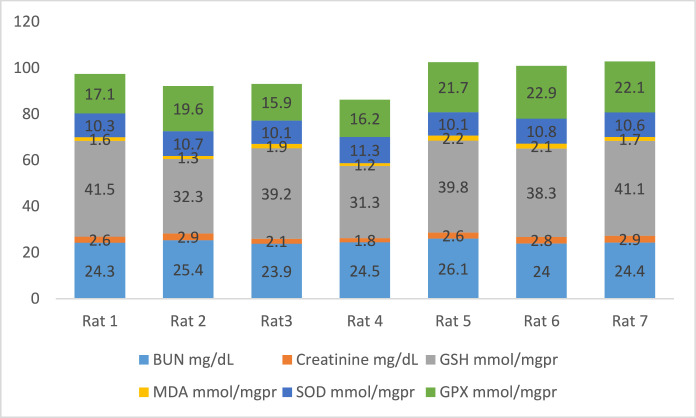
Data on kidney biochemical markers for group 6 treated with Atorvastatin (20 mg/kg body weight) and CdCl2 (2 mg/kg body weight).

**Fig. 9:**
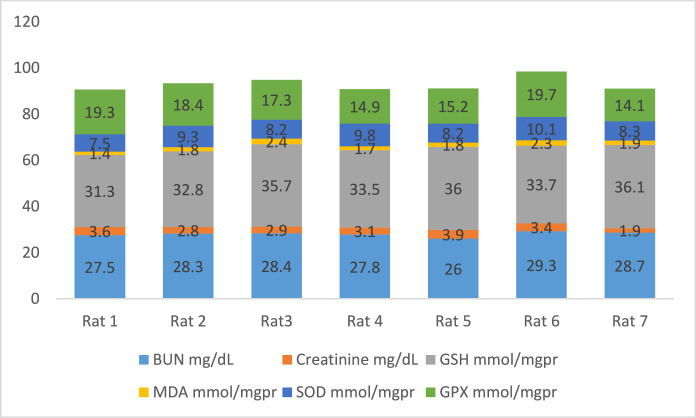
Data on kidney biochemical markers for group 7 treated with CdCl2 (3 mg/kg body weight).

**Fig. 10:**
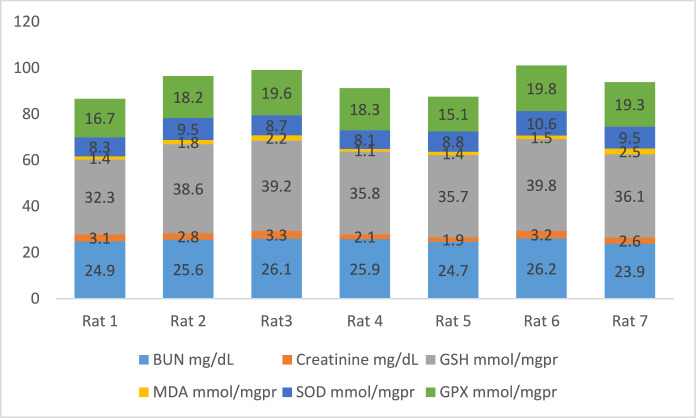
Data on kidney biochemical markers for group 8 treated with Atorvastatin (20 mg/kg body weight) and CdCl2 (3 mg/kg body weight).

Fig. 2 shows the data on the experimental periods that were conducted for the treatment and administration of Atorvastatin and cadmium chloride in Wistar rats. Data are presented on the levels of BUN, CR, GSH, MDA, SOD, and GPx for each of seven rats classified in eight groups: Group1 treated with physiologic saline (Fig. 3). Group 2 received Atorvastatin at a dosage of 20 mg/kg body weight for 15 days (Fig. 4). Group 3 treated with CdCl2 at a dosage of 1 mg/kg body weight (Fig. 5). Group 4 treated with Atorvastatin at a dosage of 20 mg/kg body weight and CdCl2 at a dosage of 1 mg/kg body weight (Fig. 6). Group 5 treated with CdCl2 at a dosage of 2 mg/kg body weight (Fig. 7). Group 6 treated with Atorvastatin at a dosage of 20 mg/kg body weight and CdCl2 at a dosage of 2 mg/kg body weight (Fig. 8). Group 7 treated with CdCl2 at a dosage of 3 mg/kg body weight (Fig. 9). Group 8: treated with Atorvastatin at a dosage of 20mg/kg body weight and CdCl2 at a dosage of 3 mg/kg body weight (Fig 10).

**Fig. 11:**
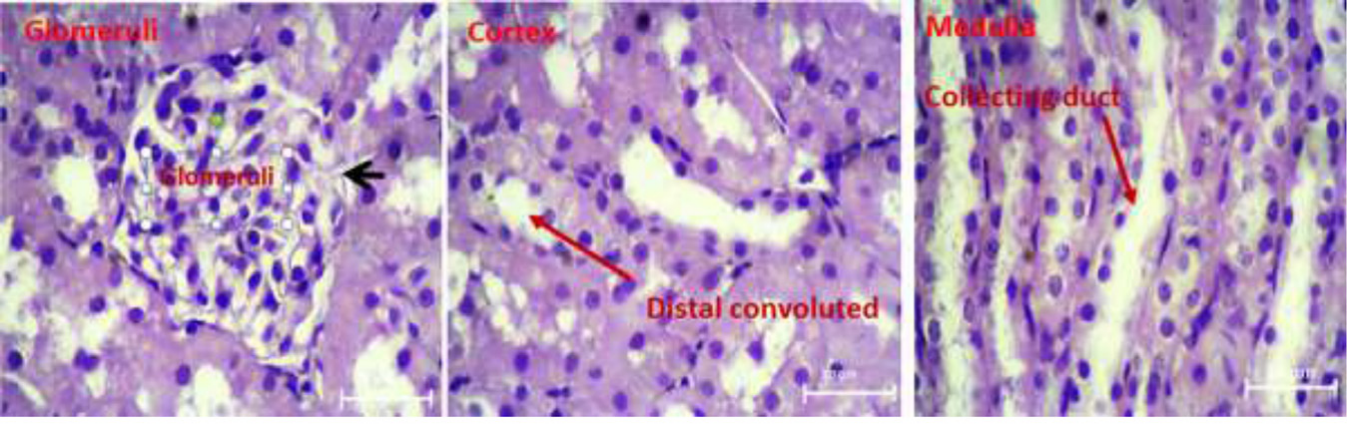
Light micrographs of rat kidney tissue of the control group treated with normal saline (x400).

**Fig. 12:**
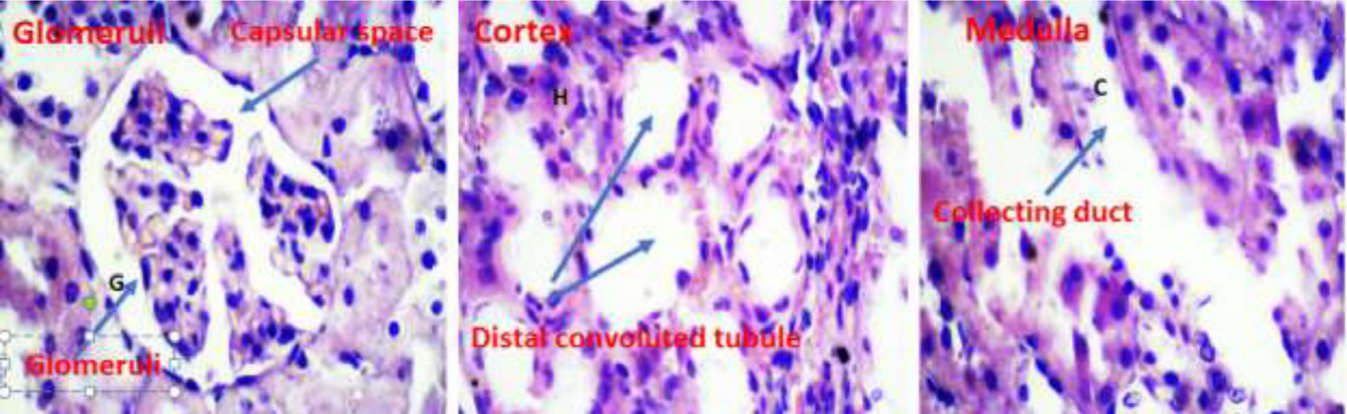
Light micrographs of rat kidney treated with CdCl2 showing renal damage: degeneration of glomeruli (G), hemorrhage (H), deposited epithelial cells in collector duct (C).(x400).

**Fig. 13:**
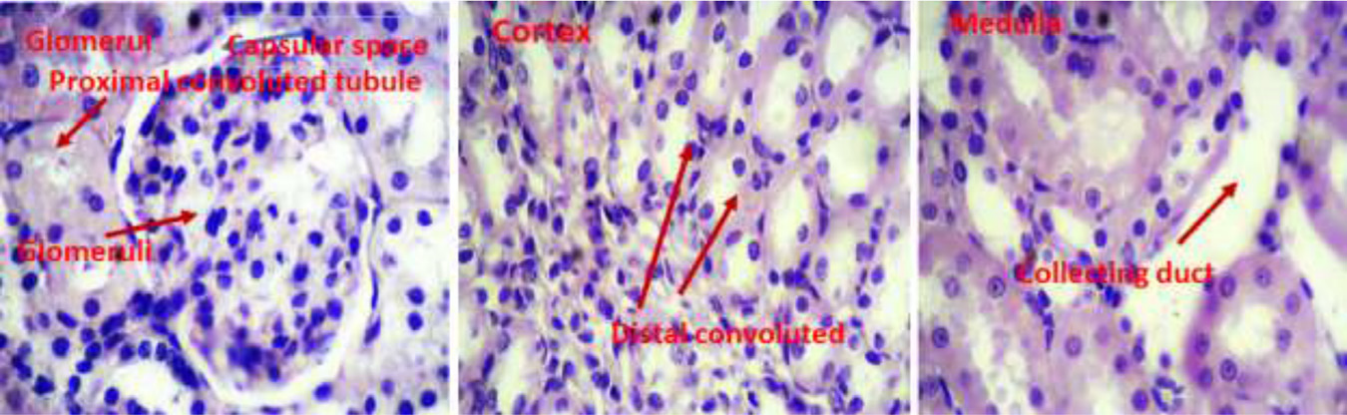
Light micrographs of rat kidney structure pretreated with 20 mg/kg AT 30 min prior to the administration of CdCl2. AT protective effect on injury in the kidney tissue, exhibiting normal kidney tissue structure with glomeruli, renal distal convoluted, and collecting duct.

Light micrographs of kidney sections of rats following treatment periods with normal saline, cadmium chloride, and pretreatment with Atorvastatin are shown in Fig. 11, Fig. 12, and Fig. 13 respectively. Histopathological analysis of the renal tissues focused on changes in glomeruli, Cortex and medullary segments.

Detailed data on the Tukey's comparisons of biochemical parameters (BUN, creatinine, GSH, MDA, SOD, and GPx) in the treatment groups of cadmium chloride-induced nephrotoxic rats with or without saline and Atorvastatin treatments are presented in the Excel data file 1 available at Mendeley repository mentioned in the above specifications table.

Results of raw data on the measurements of each rat in the experimental treatment groups are presented in the Excel data file 2 available at Mendeley repository.

## 2 Experimental design, materials and methods

### 2.1 Ethical considerations

ll animal experiments in this study complied with the ARRIVE guidelines (Animal Research: Reporting of In Vivo Experiments) and were conducted strictly in accordance with the protocols and guidelines of animal acts as proposed by the National Institutes of Health guide for the care and use of Laboratory animals (NIH Publications No. 8023, revised 1978).This research was confirmed by the institutional Ethical Review Board of Semnan University of Medical Sciences (Ref: IR.SEMUMS.REC.1395.177).

### 2.2 Materials

AT was purchased from Tehran Chemie pharmaceutical Co. Cadmium chloride (CdCl2) was purchased from Merck (Darmstadt, Germany). The reagents and chemicals of analytical grade or high purity were used for the experimental tests. GPX, SOD, GSH, and MDA were assayed using Rat GPX ELISA Kit (Cat No: ZB-GPX-96A), Rat SOD ELISA Kit (Cat No: ZB-SOD-96A), Rat GSH ELISA Kit (Cat No: ZB-GSH-96A), and Rat MDA ELISA Kit (Cat No:ZB-MDA-96A), respectively according to manufacturer instructions. ELISA kits obtained from Zellbio Germany. BUN and CR in the kidney tissues were detected as instructed by Kits obtained from Pars Azmun Pharmaceutical, Tehran, Iran.

### 2.3 Experimental rats and design

Fifty six adult Wistar male rats weighing between 200 g and 220 g were used in this study. The animals were housed in metal cages, placed 7 rats in each cage under hygienic and standard conditions and temperature maintained at 22 ± 2 °C and 12 h light/dark cycles with free access to rodent food pellets and water. Prior to experiments, rats were acclimatized and all treatments were started after one week.

Cadmium chloride at doses of 1, 2, and 3 mg/kg was dissolved in distilled water and was applied at a volume of 2 ml/kg intraperitoneally. The selected dosages of cadmium chloride were based on prior experiments on toxicity data in animals, where reported the acute intoxication of intraperitoneal injection of various doses of cadmium chloride at 1-3 mg/kg of body weight. These doses indicated biochemical and histopathological dysfunctions following short-term cadmium chloride administration, resulted in adverse effects in kidney [1,2].

Atorvastatin (dose of 20 mg/kg) [3,4] dissolved in the physiologic saline and was administered by an gastric tube (gastric gavages) at a volume of 4 ml/kg. In the previous study statins at a dose of 20 mg/kg revealed potential therapeutic effects against inflammatory diseases in liver and kidney tissues [5].

Cadmium chloride or its vehicle (normal saline) was used 30 min after the gavage administration of AT or its vehicle. Treatments were within the following timeline: Rats were treated with intra-gastric gavage of saline, AT, or vehicle of AT for 15 days, which started 7 days before intraperitoneal injection of cadmium chloride or its vehicle. On day 16 samples were collected for biochemical and histological analyses.

### 2.4 Rat groupings and treatment

The wistar rats were randomly allocated into eight groups of seven rats in each group. The first group of rats received physiologic saline. The second group treated with oral gavages a dose of 20 mg/ kg /day AT for 15 days. The third, fifth, and seventh groups received cadmium chloride with doses of 1, 2 and 3 mg/kg intraperitoneal (i.p.), while the fourth, sixth, and eighth groups pretreated with oral gavages containing 20 mg AT/kg body weight, 30 minutes prior to the intraperitoneal (i.p.) administration of cadmium chloride at doses of 1, 2 and 3 mg/kg (Excel data file 2 available at mendeley repository). All groups were treated with cadmium chloride for eight consecutive days from day 8 to day 15.

Twenty-four hours after the last treatment, all rats were anesthetized with sodium pentobarbital (50 mg/kg). Blood samples were taken from the heart of animals, and immediately thereafter kidneys were removed, dissected, and washed using normal saline solution. Parts of kidney tissue were removed, fixed and processed for light microscopy, using haematoxylin and eosin (H&E) staining technique. A segment of 0.5 × 0.5 cm^2^ of another half was used for the detection of MDA, GSH, SOD, and GPx.

### 2.5 Biochemical analyses

Data collected on cadmium chloride-induced nephrotoxicity by measuring Blood Urea Nitrogen (BUN), and Creatinine (CR) levels in serum. The levels of Malondialdehyde (MDA), Superoxide Dismutase (SOD), Glutathione Peroxidase (GPx) and Glutathione (GSH) in kidney tissue are commonly used to assess oxidative stress. While other enzymatic markers such as N-acetyl-ß-D-glucosaminidase (NAG), ß2G may also be used to estimate renal toxicity, the kits for the above biomarkers were available for the present experiment.

Kidneys tissue was homogenized in phosphate buffered saline, then centrifuged at 800 × g for 10 min at 4 °C and maintained in -80 °C for oxidative stress markers assessment [6]. Blood samples were collected from the heart of animals and the serum was obtained by centrifugation at 3000 rpm for 20 min and stored at −20 °C until the measurement of parameters. Serum concentration of BUN and CR and renal tissues concentration of MDA, GSH, GPx, and SOD were determined according to the related kits instructions. The concentration of Serum BUN was determined colorimetrically using related kits (Pars Azmoon company, Tehran, Iran) according to the method described and Serum Cr was measured by Jaffe's method, using a Commercial Kit (Pars Azmoon Co., Tehran, Iran) [7]. Concentrations of MDA, GSH, GPx, and SOD in kidney tissues were determined as per the instructions of related kits from ZellBio GmbH, Ulm, Germany.

Kidney homogenates were used to evaluate lipid peroxidation by thiobarbituric acid response to MDA Assay Kit (ZellBio GmbH, Ulm, Germany). A combination of homogenate with trichloroacetic acid produces a reactive thiobarbituric acid compound, which is a red complex. The response of the produced red color of the compound was determined at 532 nm using a Nanodrape. This method determines the MDA level with sensitivity equal to 0.1 µM [8]. SOD activity in kidney tissue determined using SOD assay kit (Zellbio Co). In this method, an enzymatic reaction converts superoxide anion to hydrogen peroxide and oxygen. To determine serum GSH and GPx levels, commercial chemical colorimetric assay kits (ZellBio GmbH, Ulm, Germany) were used through colorimetrically method at 412 nm wavelengths. The assay sensitivity was 0.1 mM [9]. GPX activity unit was determined by the amount of the sample that will catalyze 1 μmole GSH to GSSG in one minute. The protein level of in supernatants was assayed by the Bradford method using standard bovine serum albumin at 560 nm [10].

### 2.6 Histological examinations

Following fixing removed kidney tissues in formalin, a 10 μm section was taken from each tissue block. After making and drying slides, they were stained with haematoxylin and eosin (H&E) method. The criteria for cell injury included nuclear dilation, loss of staining capacity and obvious cellular swelling. In the surface of each slide, five histological fields were randomly selected and photographed under 4 × 100 magnification and evaluated by one pathologist and histologist familiar with experimental procedure.

### 2.7 Statistical data analyses

The data were analyzed using Graphpad Prism 8 computer programs. At first, all of data were analyzed by one-way analysis of variance (ANOVA). Secondly, Tukey's multiple comparisons tests were performed to compare the means of all groups to the mean of every other group. Results were presented as mean ± SE and Probability value of *P*<0.05 was determined to be statistically significant.

## Ethics statement

All the experimental tests on animals in this study complied with the ARRIVE guidelines (Animal Research: Reporting of In Vivo Experiments) and were carried out in accordance with the National Institutes of Health guide for the care and use of Laboratory animals (NIH Publications No. 8023, revised 1978). This experimental study was approved by Ethical Review Board of Semnan University of Medical Sciences (Ref: IR.SEMUMS.REC.1395.177).

## Credit Author Statement

AD, EK, ZG, AG, SY contributed to Conceptualization; Data curation; Formal analysis; Investigation; Methodology; Project administration; Resources; Software; Supervision; Validation; Visualization; Writing - original draft; review and editing.

## Declaration of Competing Interest

The authors declare that they have no known competing financial interests or personal relationships which have, or could be perceived to have, influenced the work reported in this data article.

## Data Availability

Raw and analyzed data of histological alterations and biochemical markers in rat kidney exposed to Cadmium and treated with Atorvastatin (Original data) (Mendeley Data).
